# Validation of a Novel Patient Specific CT-Morphometric Technique for Quantifying Bone Graft Resorption Following the Latarjet Procedure

**DOI:** 10.3390/jcm11195514

**Published:** 2022-09-20

**Authors:** Fraser W. Francis-Pester, Manuel Waltenspül, Karl Wieser, Greg Hoy, Eugene T. Ek, David C. Ackland, Lukas Ernstbrunner

**Affiliations:** 1 ANU Medical School, Australian National University (ANU), Canberra, ACT 2601, Australia; 2 Department of Orthopaedics, Balgrist University Hospital, University of Zurich, 8008 Zurich, Switzerland; 3 Melbourne Orthopaedic Group, Windsor, VIC 3181, Australia; 4 Department of Biomedical Engineering, University of Melbourne, Parkville, VIC 3010, Australia; 5 Department of Orthopaedic Surgery, Royal Melbourne Hospital, Parkville, VIC 3050, Australia

**Keywords:** anterior instability, Latarjet, graft resorption, computed tomography (CT), 3D assessment, morphometric analysis

## Abstract

Bone graft resorption following the Latarjet procedure has received considerable concern. Current methods quantifying bone graft resorption rely on two-dimensional (2D) CT-scans or three-dimensional (3D) techniques, which do not represent the whole graft volume/resorption (i.e., 2D assessment) or expose patients to additional radiation (i.e., 3D assessment) as this technique relies on early postoperative CT-scans. The aim of the present study was to develop and validate a patient-specific, CT-morphometric technique combining image registration with 3D CT-reconstruction to quantify bone graft resorption following the Latarjet procedure for recurrent anterior shoulder instability. Pre-operative and final follow-up CT-scans were segmented to digitally reconstruct 3D scapula geometries. A virtual Latarjet procedure was then conducted to model the timepoint-0 graft volume, which was compared with the final follow-up graft volume. Graft resorption at final follow-up was highly correlated to the 2D gold standard-technique by Zhu (Kendall tau coefficient = 0.73; *p* < 0.001). The new technique was also found to have excellent inter- and intra-rater reliability (ICC values, 0.931 and 0.991; both *p* < 0.001). The main finding of this study is that the technique presented is a valid and reliable method that provides the advantage of 3D-assessment of graft resorption at long-term follow-up without the need of an early postoperative CT-scan.

## 1. Introduction

The Latarjet procedure has become one of the most commonly used bone block procedures for the treatment of recurrent anterior shoulder instability [1,2,3]. Though the method has undergone various modifications, its longevity is owed to its reliable clinical outcomes, with successful stabilization of the shoulder and low rates of recurrent instability [4,5,6,7,8,9]. 

Despite excellent clinical outcomes, the Latarjet procedure has a number of complications including ongoing apprehension, recurrent instability and complications related to the metalware [6,7,10]. The complication that has received major concern is that of substantial coracoid graft resorption, which has been suggested to contribute to failure of the surgery [11,12]. While resorption of the bone graft in the Latarjet procedure is an accepted phenomenon, which patients may be at risk of excessive bone resorption and where graft osteolysis may be concentrated remains to be confidently resolved [11,13,14]. It has also been suggested that patients with minimal pre-operative glenoid bone loss undergo more significant graft resorption following the procedure than patients with large pre-operative glenoid bone loss [14]. 

Zhu et al. (2015) developed a classification system to grade the severity of graft resorption following the open Latarjet procedure [11]. Rather than quantify resorption by assessing the whole volume of the graft, the system uses 2-dimensional (2D) axial CT scans to assess focal osteolysis around the two screws in the graft. While the system was demonstrated to have high inter- and intra-rater reliability for assessing the severity of graft resorption, the technique provided no morphologic or volumetric information. Bone remodelling is a three-dimensional process that two-dimensional techniques fail to appreciate as they include no information on global graft morphology which is a prerequisite to assess where graft osteolysis may be concentrated and conversely, where it may be conserved.

Three-dimensional techniques for quantifying bone graft volume have been used by various authors to measure Latarjet-graft osteolysis at short-term follow-up. Di Giacomo et al. (2011) used a 3-dimensional (3D) CT-based technique to reconstruct patient’s scapulas 3 days after surgery and after a mean of 1.5 years [12]. Haeni et al. (2015) also developed a 3D volumetric technique, segmenting sagittal CT scans at 6 weeks and 6 months after surgery to quantify graft resorption [13]. The 3D technique described by Kee et al. (2018) assessed graft osteolysis by measuring glenoid surface area at around 6 months and after 2.5 years [15]. These techniques have several limitations. First, they require three shoulder CT scans, thereby exposing patients to extensive radiation within one to two years around the actual procedure. Second, the techniques have measured graft resorption over time intervals where the coracoid graft may be easily distinguished from the glenoid rim. They may be more limited for assessing long-term graft volume when it has integrated into the glenoid and undergone significant remodelling. The techniques also only reconstructs the patient’s scapulas using a single CT imaging plane which may be insufficient to accurately reconstruct the graft, especially considering the significant metal artifact from the screws. These limitations may be overcome by combining image registration, a method of superimposing two images of the same structure at different time points, with three-dimensional digital segmentation and reconstruction, a technique that has been widely used in other specialties such as maxillofacial surgery [16,17]. This so called ‘subtractive method’, registers pre- and post-intervention CT scans to produce three-dimensional- reconstructions that allow for accurate calculation of the initial defect and monitoring of graft volume after corrective surgery [16].

It was therefore the aim of the current study to develop and validate a patient-specific, CT-morphometric technique combining image registration with 3D CT reconstruction to quantify coracoid bone graft resorption at long-term follow-up in patients that have undergone an open Latarjet procedure for recurrent anterior shoulder instability. It was hypothesized that CT-morphometric measurements of graft resorption at long-term follow-up will positively correlate with the Zhu classification (i.e., as Zhu resorption grade increase, graft resorption will increase) and that this new technique has good inter- and intra-rater reliability. 

## 2. Materials and Methods

### 2.1. Ethical Approval

This retrospective study was approved by the Cantonal Ethics Committee (No. 2018-01929). All patients included gave written consent for the purpose of this study.

### 2.2. Participants and Image Acquisition

From January 2008 to December 2013, 105 consecutive patients older than 18 years of age were treated with an open Latarjet procedure for recurrent anterior instability. Patients with (1) previous surgery on the affected side; (2) revision of the Latarjet; (3) multidirectional instability; (4) contralateral instability; (5) medical conditions affecting shoulder function (e.g., rheumatoid arthritis); (6) advanced dislocation arthropathy; and (7) incomplete imaging were excluded from this study. 

The reviewed cohort consisted thus of 31 patients (29 male, 2 female) with a mean age of 37 years (range, 28–50) at the time of the primary open Latarjet procedure. Patients were interviewed and examined at a mean follow-up of 8.5 (range, 7.5–10.5) years. Decision for a primary open Latarjet procedure was based on preoperative glenoid bone loss >15% [18], and/or if the patients had a high physical activity level or were heavy laborers.

Patients received a CT-scan of the involved shoulder before surgery and after a mean of 8.5 years postoperatively. Scout images were used to confirm the CT was in the plane of the scapula body for all CT scans assessed. All CT scans were obtained using a 64-slice Somatom Sensation 64 CT scanner (Siemens, Erlangen, Germany) with a slice thickness of 0.75mm and a pitch of 0.8. These CT-scans were assessed for bone graft resorption according to the Zhu classification [11], and for CT-morphometric volume measurements as described below.

### 2.3. Surgical Technique 

The open Latarjet procedure was performed according to the Walch refinement of the original technique [1,19]. A deltopectoral approach was performed and the coracoid was osteotomised at its base using bent chisels. The subscapularis was split with a horizontal cut slightly below its mid-level to expose the glenoid neck and the bone graft was fit flush to the glenoid in the 2 to 5 o’clock position. The graft was fixed with two 4.5 mm AO malleolar screws (Synthes, West Chester, PA, USA). The free edge of the coracoacromial ligament was sutured to the medial aspect of the incised capsule. Following surgery, patients’ shoulders were immobilized for four weeks in a sling, with restrictions on bicep movement and combined abduction and external rotation restricted for 6 weeks. 

### 2.4. Assessment of Bone Graft Resorption according to the Zhu Classification

**Figure 1:**
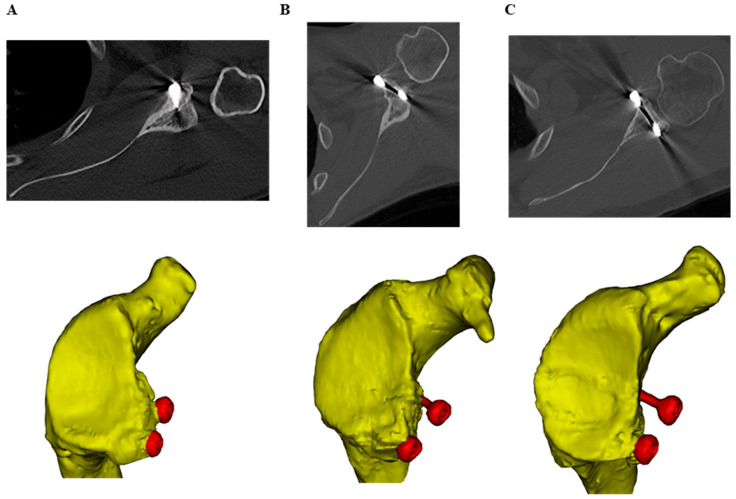
Resorption grades on the final follow-up axial CT scans, as defined by Zhu et al. (2015) [11], with the corresponding three-dimensional scapula geometries (yellow) and screws (red) generated by segmentation of CT scans of three different participants. (**A**) Grade I (minor resorption), (**B**) Grade II (major resorption) and (**C**) Grade III resorption (total resorption).

Bone graft resorption was assessed as per the Zhu classification [11] for each patient on their computed tomography scans obtained at final follow-up. The classification system defines bone resorption on the axial CT scans of patients in terms of four grades: Grade 0 (no resorption) where the head of the screw remains buried in the graft; Grade I (minor resorption) where only the screw head is exposed outside the bone graft; Grade II (major resorption), where part of the head and the screw shaft is exposed though some bone graft remains on the glenoid neck; and Grade III (total resorption), where the screw head and screw shaft are completely exposed and no graft remains on the glenoid neck (Figure 1). 

As per the classification system, bone resorption was evaluated around both screws, with the highest grade between the two being used to classify the patients’ overall bone resorption (i.e., if one screw had grade I resorption and the other had grade III, the patient was classified as having grade III resorption).

### 2.5. CT Morphometric Analysis 

**Figure 2:**
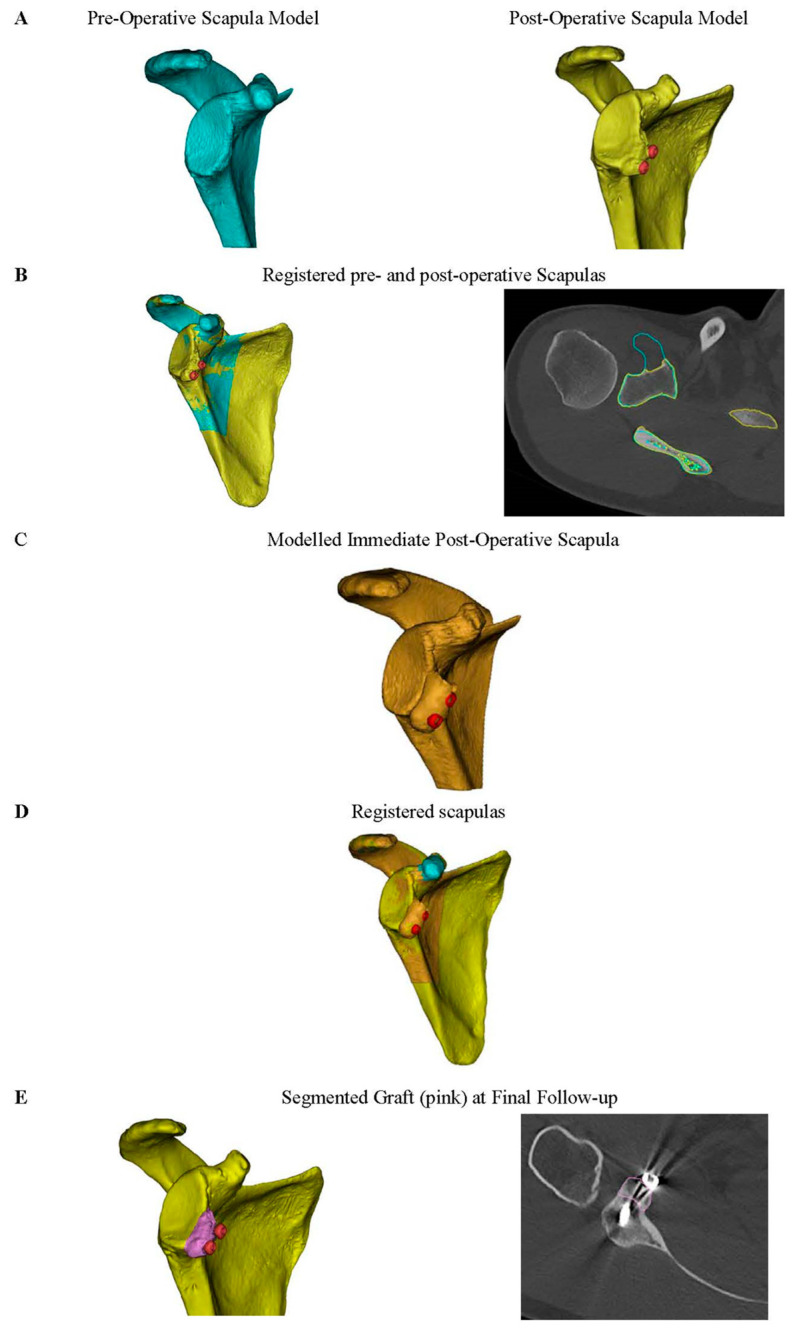
(**A**) Computed tomography (CT) scans were digitally segmented using Mimics (Materialise, Belgium) to reconstruct three-dimensional geometries of each patient’s pre-operative (blue) and post-operative (yellow) scapula at final follow-up (mean 8.5 years). (**B**) These geometries were registered to determine where the coracoid graft was taken from during the Latarjet procedure. (**C**) The coracoid graft was virtually osteotomised at this point and fit to the glenoid of the pre-operative scapula, to produce a third scapula geometry equivalent to a modelled immediate post-operative scapula (orange). (**D**) To help distinguish the remodeled graft at final follow-up from the glenoid, the registered geometry of the pre-operative, post-operative and modelled immediate post-operative scapula were used. (**E**) This facilitated accurate segmentation of the graft from the glenoid at final follow-up (pink) and accurate measurement of its volume.

The pre-operative and final follow-up CT scans of each patient were manually segmented to digitally reconstruct the entire three-dimensional geometry of the scapulas (Mimics, Materialise, Belgium). The pre-operative scapula geometry was then registered with the post-operative scapula, and the site of the coracoid process osteotomy performed for the Latarjet procedure was identified and digitally marked on the pre-operative scan (Figure 2A,B). 

A virtual osteotomy was then performed on the pre-operative scapula geometry at this site in order to reproduce the 3D geometry of the bone graft used during the initial Latarjet procedure (i.e., timepoint zero graft geometry). The modeled coracoid graft was then virtually placed on the glenoid of the pre-operative scapula, to produce a third scapula geometry equivalent to an immediate post-operative scapula (Figure 2C). The inferior region of the actual Latarjet graft was used as a reference for exact positioning of the modeled graft on the glenoid. Previous studies have demonstrated that the inferior region of the coracoid graft undergoes minimal bone resorption following the Latarjet procedure [12,13,14]. The resulting graft model was used to represent the immediate (timepoint zero) post-operative graft volume.

To help distinguish the graft at final follow-up from the glenoid, the registered geometry of the pre-operative scapula and of the post-operative scapula were used. The actual Latarjet graft at final follow-up was then digitally segmented and identified from the scapula (Figure 2D). To reduce the metal artifact, the screws were segmented separately to the graft using a high threshold, thus helping to distinguish them from the graft. The screw heads were removed from the final bone graft to ensure they did not contribute to the overall volume. The volume of the bone graft at final follow-up was then recorded and compared to that of the modeled graft, thus determining 3D graft resorption at long-term follow-up (Figure 2E).

CT-scans segmentation, reconstruction and graft identification were performed by one fellowship-trained upper limb surgeon (LE) and one medical student (FFP).

### 2.6. Statistical Analysis

Normal distribution was tested with the Shapiro–Wilk test. Bivariate correlation between Zhu resorption grade and CT-morphometric graft resorption was assessed using the Kendall tau correlation coefficient, which allows correlation between ordinal (Zhu grading) and continuous variables (graft resorption). Significance level was set at *p* < 0.05. Statistical analysis was performed using the Statistical Package Social Science (SPSS) (PASW Statistics 18, SPSS Inc., Chicago, IL, USA).

Inter- and intra-observer reliability was assessed for the CT-morphometric volume measurements by means of the Intraclass Correlation Coefficient (ICC) for absolute agreement, with 1 indicating perfect reliability.

## 3. Results

According to the Zhu classification, all patients (n = 31, 100%) demonstrated bone graft resorption. The majority of patients displayed Grade II resorption (n = 18, 61%) of the coracoid bone graft, followed by Grade I (n = 8, 23%), with only 5 (16%) of patients showing Grade III resorption (Table 1). The mean modeled graft volume at timepoint zero was 2931 mm^3^ compared to 2010 mm^3^ at final follow-up, which represented an overall graft resorption of 30.9% (921 mm^3^).

Bone graft resorption was strongly correlated with the Zhu resorption grade, with a Kendall tau correlation coefficient 0.73 (95% CI [0.59, 0.83], *p* < 0.001). The technique demonstrated excellent inter-rater reliability with an ICC value of 0.930 (95% CI [0.75–0.98], *p* < 0.001). Intra-rater reliability, was also excellent with an ICC value of 0.991 (95% CI [0.963, 0.998], *p* < 0.001).

## 4. Discussion

The objective of the present study was to develop a new patient-specific CT-based morphometric technique, utilizing both image registration and 3D reconstruction, to measure long-term coracoid graft bone resorption in patients who have undergone the Latarjet procedure for recurrent anterior shoulder instability. The hypothesis that measuring 3D bone graft resorption would positively correlate with the 2D Zhu classification was supported by the results of the study. The study also demonstrated that our technique has a high reliability in calculating bone graft resorption, even after a long-term remodelling process, in terms of excellent inter- and intra-rater reliability. To the authors knowledge, this is the first technique to combine CT image registration with 3D segmentation and reconstruction to measure graft volume and resorption at long-term follow-up after the Latarjet procedure. 

A major advantage of the new technique is the reduced radiation dose to patients compared to previous 3D techniques. As such, the techniques described in the literature all require a third CT scan taken immediately post-operatively in addition to the standard pre-operative and final follow-up CT scans. Our technique overcomes the need for an immediate postoperative CT scan by modelling the scapula geometry equivalent to an immediate post-operative scapula based on registration of preoperative and final follow-up geometries of the scapula. Another advantage of our 3D measurement technique is that it directly measures volume and graft resorption. This allows for global graft morphology to be appreciated and for accurate assessment of where focal graft osteolysis may be concentrated and conversely, where it may be conserved. As such, previous studies showed that osteolysis is concentrated in different regions of the graft, which was determined by 3D-based techniques [12,13,14]. Di Giacomo et al. found that resorption was concentrated in the superficial and medial regions of the graft, while Haeni et al. [13] found that resorption was greatest in the superior half of the graft. The current 3D measurement technique, as well as quantifying focal graft resorption, also allows for the graft to be better distinguished from the glenoid at all time-points. The mean follow-up time in our study was 8.5 years and in certain cases complete remodeling/integration of the graft into glenoid had occurred, making segmentation of the actual bone graft difficult. As the registered geometry of the pre-operative scapula was superimposed over the post-operative geometry of the scapula, distinguishing between the actual glenoid and the bone graft was simple. Another advantage of our technique is the high reliability and ease of use. Although high-quality CT-scans are a prerequisite, the current results showed that virtual osteotomy and 3D-volumetric analysis can be reliably conducted, both with a high reliability if repeated by the same person and also with a high reliability if repeated by a different person. Although no learning-curve analysis was conducted nor the time needed to complete the measurements was analyzed, we feel that the technique is simple and quick. With the advantages of artificial intelligence-segmentation, further studies might reveal an automated method to conduct the 3D morphometric analysis proposed in our study, which would reduce time and further increase reliability.

Calculated coracoid graft resorption in our study was 31% after a mean follow-up of 9 years. No other study in the literature has reported on long-term 3D graft resorption following the Latarjet procedure. There are certain studies reporting on graft resorption at short-term follow-up, however, their results are contradictory. A previous study using a 3D CT technique, assessed that 6 months postoperatively 23.2% of the total graft volume was resorbed compared to 6 weeks postoperatively [13]. Another study using 3D CT reconstruction determined that 59.5% of the graft volume was resorbed 17 months postoperatively [12]. Part of the discrepancy may be explained by the prolonged follow-up. Over the long-term follow-up period in our study, certain patients showed almost a reversed effect in bone graft resorption, as the graft at final follow-up was close to 100% compared to the calculated graft volume. This might be due to a compensational process (Wolff’s law) as a response to ongoing (micro-)instability. However, it could also be a degenerative process and therefore formation of osteophytes that add to the final graft volume. Notwithstanding, all patients in the present study observed graft resorption in all 31 patients (100%). This incidence slightly higher than that reported in a previous study of 91%, though this discrepancy is most likely the result of the longer follow-up time in the present study (9 years compared to 1 year of follow-up) [11]. Although previously published results suggest that majority of the resorption process happens during the first postoperative year, the difference in overall resorption compared to our results may direct towards the theory that bone resorption and remodelling is an ongoing process and does not stop after the first year after the Latarjet procedure. Another limitation of the previous studies is graft geometry was mostly reconstructed from single-plane CT scans. We have segmented the coracoid graft geometry using axial, coronal and sagittal CT scans, thus improving the accuracy of coracoid graft segmentation and reconstruction.

There are limitations of the technique for consideration. First, the current technique segments the coracoid graft from where it was cut during surgery and does not account for minor macroscopic remodelling during surgery (i.e., trimming graft to cancellous bone) to create a bleeding surface area. Second, the study does not include a control group which, with early post-operative scans, would allow for actual intraoperative graft loss to be quantified and accounted for. A further limitation of the study is the low number of patients, particularly in the grade I and grade III resorption groups. However, the correlation coefficients were high with high significance, proving the validity of our technique. Last, high-quality CT-scans are a prerequisite for the technique, which are expensive and not available in every hospital.

## 5. Conclusions

This study describes a novel CT morphometric technique that uses combined image registration and 3D reconstruction to accurately measure graft volume and long-term bone resorption in patients that have undergone the Latarjet procedure. The main finding of the study is that measured 3D bone graft resorption had excellent correlation to the 2D gold standard classification by Zhu, and that the technique is highly reliable. This technique could be readily utilized in future research for the study of bone resorption at long-term follow-up and in clinical assessments of graft osteolysis following the Latarjet procedure, thus guiding surgeons regarding possible revision surgery when faced with significant resorption of the coracoid bone graft.

## Figures and Tables

**Table 1:** Mean modeled coracoid graft volume at timepoint zero (mm^3^) and mean reconstructed graft volume at final follow-up (mm^3^) stratified by resorption grade as proposed by Zhu et al. (2015) [11].

Zhu Resorption Grade	N	Mean Reconstructed Coracoid Graft Volume at Timepoint Zero (mm^3^)	SD	Mean Reconstructed Graft Volume at Final Follow-Up (mm^3^)	SD	Bone Graft Resorption (%)
Grade 0	0	-	-	-	-	-
Grade I	8	2724	492	2369	440	−12.3%
Grade II	18	3022	495	2068	410	−31.5%
Grade III	5	2933	447	1228	415	−58.3%
Total	31	2931	488	2010	549	−30.9%

## Data Availability

Not applicable.
